# Perforation gastrique néonatale spontanée: à propos d'un cas

**DOI:** 10.11604/pamj.2015.21.61.5560

**Published:** 2015-05-26

**Authors:** Abdelhalim Naji, Yahya Elkarout, Noufissa Benajiba

**Affiliations:** 1 Service de Pédiatrie, CHU Mohammed VI, Université Mohammed Premier, Oujda, Maroc; 2 Service de Chirurgie Infantile, Centre Hospitalier Régional Al Fârâbî, Oujda, Maroc

**Keywords:** Nouveau-né, détresse abdominale, abdomen sans préparation, perforation gastrique spontanée, newborn, abdominal distress, abdominal radiography without preparation, Spontaneous gastric perforation

## Abstract

La perforation gastrique néonatale spontanée est rare. Nous rapportons un cas survenu chez un nouveau-né issu d'une grossesse et une naissance sans anomalies, et qui a présenté au deuxième jour de sa vie brutalement une distension abdominale importante, suivie d'une détresse respiratoire nécessitant des mesures de réanimation brèves. La radiographie de l'abdomen sans préparation montrait un pneumopéritoine massif, la laparotomie trouvait une perforation au niveau de l'antre gastrique de 2cm, qui était suturée en un plan. Les suites opératoires étaient simples. L’évolution des perforations gastriques spontanées survenant chez le nouveau-né est habituellement favorable. Sous réserve d'un diagnostic et prise en charge précoce.

## Introduction

La perforation gastrique du nouveau-né est une affection rare, au pronostic encore grave. Le taux élevé de mortalité chez ces patients peut être améliorée par un diagnostic précoce et de réanimation rapide qui nécessite une prise en charge chirurgicale. Notre objectif est de rendre service aux cliniciens mis en présence d'une situation analogue. Nous rapportons ici un cas de perforation gastrique idiopathique survenus chez un nouveau-né à terme.

## Patient et observation

**Figure 1:**
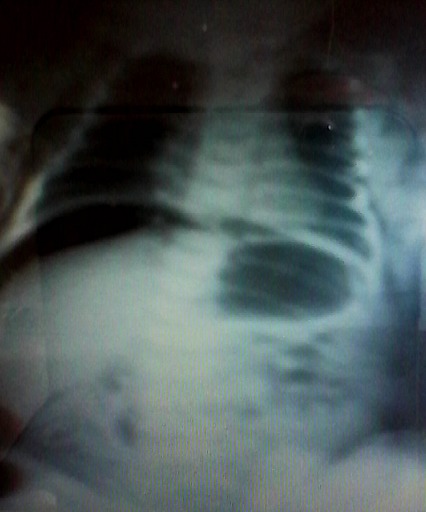
Radiographie de l'abdomen sans préparation montrant un pneumopéritoine massif évoquant La perforation d'un viscère creux

**Figure 2:**
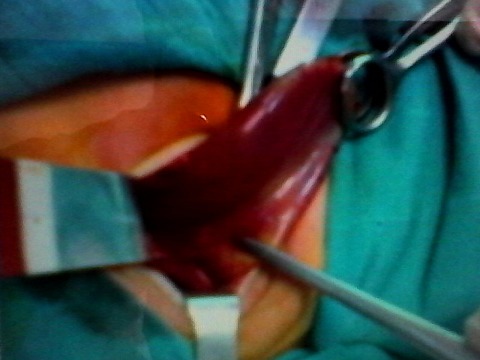
Montrant une perforation au niveau de l'antre gastrique de 2cm lors d'une laparotomie

Un nouveau-né d'un jour, de sexe féminin, a été admise d'urgence pour une importante distension abdominale avec détresse respiratoire. Cet enfant était née à terme, par voie basse, à la suite d'une grossesse normale, avec un poids de naissance de 3 kg. Aucune mesure de réanimation n'avait été nécessaire lors de l'accouchement. Le placenta et le cordon ombilical étaient normaux et le liquide amniotique clair. L'allaitement a été débuté dès la naissance et l’émission de méconium s'est faite dans les premières 24 heures. À l’âge de trois jours est apparue une distension abdominale, d'installation rapide, qui devenait en quelques heures massive et était accompagnée de vomissements alimentaires puis bilieux. À ces signes digestifs s'associaient des signes respiratoires avec tachypnée à 50 c/minute, tirage intercostal et sus-sternal, battement des ailes du nez et cyanose modérée des extrémités. La fréquence cardiaque était de 150 c/minute et les pouls périphériques étaient à peine perceptibles. Ce nouveau-né était prostré, apathique avec un abdomen très distendu, sans aucun bruit hydroaérique à l'auscultation abdominale et présentait un état de détresse respiratoire avec des signes d'encombrement bronchique à l'auscultation pulmonaire. La radiographie de l'abdomen sans préparation mettait en évidence un pneumopéritoine massif évoquant fortement une perforation d'un viscère creux (Figure 1). Une oxygénothérapie par sonde nasale simple, une mise en place de sonde nasogastrique, une rééquilibration hydro électrolytique et une antibiothérapie associant ceftriaxone; ampicilline et gentamycine ont été prescrites et soulageaient légèrement l'enfant. Une laparotomie exploratrice était indiquée, trouvait une perforation gastrique en regard de la grande courbure (Figure 2), qui était suturée en un plan. Les suites opératoires étaient simples. L’évolution des perforations gastriques spontanées survenant chez le nouveau-né est habituellement favorable sous réserve d'un diagnostic et d'une prise en charge précoces.

## Discussion

La perforation gastrique spontanée est rare chez le nouveau-né né à terme et représente 10 à 16% des perforations gastro-intestinales néonatales. Depuis la première description de Siebold en 1825, plus de 300 cas ont été rapportés dans la littérature. La qualification de «spontanée» correspond à une entité propre et sont exclues les perforations gastriques associées à une obstruction distale. L’âge habituel de survenue se situe entre deux et sept jours et il existe une prédilection pour la race noire et le sexe masculin [1]. Plusieurs mécanismes sont avancés dans la genèse de la perforation avec des aspects anatomopathologiques particuliers selon l’étiologie. Ainsi, ont été rapportées les perforations d'origine congénitale par agénésie de la musculature gastrique occasionnant des lésions à type de déchirure linéaire au niveau de la grande courbure; puis les perforations d'origine ischémique (souffrance néonatale, embole septique), d'origine mécanique(distension gastrique après ventilation au masque trop appuyée, sonde gastrique perforant l'estomac), d'origine médicamenteuse (corticoïdes, indométacine dans la communication inter-auriculaire) ou encore d'origine fonctionnelle (affection neurologique, atonie gastrique, spasme pyloriqueen cas de stress néonatal) entraînant des perforations punctiformes de la paroi gastrique antérieure ou postérieure. Par ailleurs, plusieurs facteurs de risque sont associés à l'affection: la prématurité, le faible poids de naissance, l'exsanguino-transfusion, la rupture prématurée des membranes, la toxémie gravidique, l'accouchement par le siège, le diabète maternel, le placenta prævia, l'infection amniotique ou encore la césarienne [2]. Aucun de ces mécanismes ou facteurs de risque ne semble être en cause dans notre observation et l'origine reste indéterminée.

Le tableau clinique de la perforation gastrique néonatale est assez caractéristique. En effet, lors des premiers jours alors que l'alimentation et l’émission de méconium sont normales, ce qui élimine une cause obstructive, surviennent brutalement une distension abdominale, des vomissements et des troubles respiratoires qui évoluent en peu de temps vers une détresse respiratoire. L’état général est vite altéré et la radiographie de l'abdomen sans préparation montre un pneumopéritoine massif caractéristique. Pour le diagnostic différentiel, il faudra éliminer essentiellement les perforations gastriques néonatales secondaires à une cause obstructive sous-jacente (atrésie intestinale, iléus méconial, maladie de Hirschsprung, etc.), l'entérocolite nécrosante, les occlusions intestinales néonatales, les perforations, les pneumopéritoines sans brèche digestive accompagnant un pneumothorax ou un pneumomédiastin, exceptionnellement une pneumatose intestinale primitive, et les pneumopéritoines idiopathiques. Sur le plan thérapeutique, certains auteurs préconisent, avant la chirurgie, une ponction abdominale de décompression pour soulager le travail respiratoire. Le traitement chirurgical consiste en une suture de la perforation qui peutpar ailleurs être multiple, ce qui justifie une exploration minutieuse. Une toilette péritonéale avec une solution salée tiède et un assèchement de la cavité abdominale permettent, comme dans notre cas, d’éviter un drainage. L’évolution est la plupart du temps favorable pour les cas diagnostiqués et traités rapidement. La mortalité reste cependant élevée en cas de retard de la prise en charge, de grande prématurité ou de sepsis sévère associé [3].

## Conclusion

La perforation gastrique chez le nouveau-né est une urgence chirurgicale rare mais grave qui met en jeu le pronostic vital. Le pronostic dépend de la rapidité du diagnostic et de la réanimation en pré et en postopératoire.
